# Education before Birth

**Published:** 1872-04

**Authors:** P. H. Hayes


### EDUCATION BEFORE BIRTH.

P. H. HAYES, M. D.

In Dr. Brown's "Spare Hours," we find the story of a brisk amateur student who approached Mr. Opie, the great historical painter, with the question, "Pray, sir, may I ask what you mix your colors with?" "With *brains*, sir!" was the gruff reply. A reply, it must be admitted, rather general in its character, yet it struck out the principle, that *brains* and only *brains* are success.

It will not, however, quite do to say of brains, as has been said of poets, that they are *born*, not *made*, for undoubtedly brains are both born and *made*, too; but we do owe so much to birth that old Fernelius was hardly extravagant, when he said, it is the greatest part of our earthly felicity to be well-born. The following illustrations are designed to show, not only that a general type of mind is transmissible from parent to child, but that even acquired faculties and moral dispositions, in vigorous exercise, become inborn, that in some sense and degree we are in fact educated before our birth.

In Austin's characteristics of Goethe, the author says: It has often been remarked that great and eminent men receive from their mothers, even before they see the light, half the mental dispositions and other peculiarities of character by which they are afterward distinguished. Farther on, Goethe's character is portrayed as the exact counterpart of his mother's in every thing which relates to his fine sensibility, his poetic imagination, his fondness for story-telling, and his endowment of *mother*-wit.

Mitford begins his biography of Milton in these words: John Milton, *magnum et venerabile nomen*, the son of John Milton and Sarah Castor, a woman of incomparable virtue and goodness. In Milton's Doctrine of Divorce, he is believed to allude to his own wife where he speaks of a "mute and spiritless mate, an image of earth and phlegm," and these characteristics would apparently suffice to have extinguished all traces of his genius, as seems to have been the fact in his own children.

Napoleon's mother, Letitia Romilini, was one of the most beautiful women of her time, and possessed of remarkable firmness of character. She is said to have shared with her husband the fatigues and perils of the civil war in Corsica, and to have passed much of her time on horseback, previous to the birth of the future Emperor. Here, the excitement and the work of war gave to the mother that very type of personality, which became intensified in him who worshiped no God but ambition, whose arms awed the world, and "shook its thrones, and to the earth its mitres cast."

> "Whose falchion flashed along the Nile,
> Whose hosts he led o'er Alpine snows,
> O'er Moscow's towers, that blazed awhile,
> His eagle flag unrolled and froze."

In Dr. Madden's commentary on Lord Byron, he speaks of an "hereditary taint," doubtless derived from his "unhappy tempered mother," and says again, that little is known of her early history, but quite enough of the extraordinary violence of her temper and of its effects upon her health, to justify the belief that some brain disease occasioned that degree of excitability, which is quite unparalleled in the history of any woman of a truly sane mind.

A mother, of fine endowments of head and heart, and a fine physical constitution, told me that she took into her house an invalid subject to attacks of great nervous distress, of whom she herself took the care, and by whom she was often broken of her rest at night. Her child, born about this time, was "marked," not by any outward discoloration or deformity, for which mothers anxiously look, but in its nervous system, suffering afterward, and on to mature age, as did the invalid of whom the mother had taken charge.

An eminent writer says: I know two young sisters, opposite as the poles in their tendencies and dispositions. One is impatient, fretful, revengeful, and seldom satisfied with any thing or person around her. The other is every way kind, gentle and loving in her nature. Before the birth of the first, the parents were laboring under pecuniary anxieties, and suffering wrongs that filled them with irritation and impatience. Before the birth of the second sister, these unhappy influences had passed away, the mother was calm, happy and loving, and thus the second daughter was educated before her birth by influences of an exactly opposite character.

Even our domestic animals may give us many illustrations of the law we are considering. M. De Frarière, a French physician, records the following: He says, my father had rendered some service to the Prior of the great St. Bernard, who afterwards gave him a pair of the world-renowned Alpine Spaniels. They grew to an immense size, and although very gentle, strangers were afraid of them. My father was obliged to chain them up, against which the good Prior protested, and added, if it could not be helped, we must not keep the young ones born under such circumstances. The Prior was right. The dogs born while the mother was chained up, gloomy and often angry at the children, who teased her through the palings, we were obliged to kill, on account of their ferocious disposition, while those born afterward, and when the mother was free and happy, were as gentle as lambs.

Perhaps no human power or capacity is more frequently transmitted than the talent for music. The mother of Mozart was passionately fond of music previous to his birth, but later in life, having lost all disposition for music, another son born at this time, was quite destitute of any talent of this kind. M. De Frariére says, Madame Mamo has a daughter only four years of age, who already displays a great faculty for music. It is wonderful how this little *artiste*, who has never received a single lesson, will reproduce, with her little crystal voice, all the turns and all the elegances, and the most delicate expressions of her mother's renderings.—This talent is easily accounted for. Her mother sang up to, and even on the eve of the very day, when they could print that both mother and child were doing well.

These illustrations of talents and moral dispositions transmitted, carry with them a great moral significance, and show very clearly how truly the parent is represented in the child.

The story of Hannah, her prayer and her vow, as given in the first Chapter of First Samuel, is full of natural simplicity, religious faith and poetic interest. Her profound piety gives her an intense personality, and with this she endows her unborn son. Thus was young Samuel filled with the Spirit from his birth, and ministered unto the Lord from his childhood.

St. Paul wrote to Timothy, "When I call to remembrance the unfeigned faith that is in thee, which dwelt first in thy grandmother Lois, and in thy mother Eunice." At this very moment he was in the power of the Emperor Nero, a prince in cruelty, even where cruelty had been the cool character of his nations for ages. The Bible itself represents the Romans as having stamped the nation they conquered as with a heel of iron, and without a spark of mercy for sex or age. And though history tells us that for three generations Nero's ancestors were distinguished for fierce and cruel passions, yet no man's moral sense fails to hold him responsible for his terrible character.

Bearing upon this point, George Sand makes her hero in "Mauprat" say "it would be wrong in me to claim, in order to persuade you to regard me with pity during the first few years of my life, that I was born with a noble organization, and a pure and incorruptible soul. I do not know, sir, how this may have been. It may be there are incorruptible souls, and it may be otherwise. Neither you nor any other person will ever know. There is no more important question to be solved than this. Are our propensities invincible, or is it in the power of discipline or education, to modify and perhaps even to destroy them? For my part, I dare not decide; but I have had a terrible life, gentlemen; and were I a legislator, I would cut out the tongue, or cut off the arms of any man who dared to preach or to write that the organization of an individual is fatal—that the character of a man can no more be re-created than the appetite of a tiger. God has preserved me from such a belief. We all need to be *loved* in order that the good in us may be developed, but we need to be loved each differently, one with unwearying indulgence, another with steady severity." "God is *love*." "*Love* is the fulfilling of the law."

